# The Yin and Yang of Exposure: Chemical Combinations May Explain Feminization of Wild Fish

**DOI:** 10.1289/ehp.117-a211a

**Published:** 2009-05

**Authors:** Julia R. Barrett

More than 100,000 substances occur in wastewater effluent, including an array of endocrine disruptors such as human and veterinary pharmaceuticals, natural and synthetic hormones, detergents, and industrial chemicals. Studies have linked estrogenic wastewater pollution with feminization of males in downstream fish populations. However, findings from rodent models of testicular dysgenesis syndrome, a spectrum of environmentally linked male reproductive disorders in humans, indicate that both estrogens and antiandrogens may be contributing to health effects in tandem. A new study that models exposure to both estrogenic and antiandrogenic compounds in wild fish now suggests that combinations of these compounds, rather than estrogenic compounds alone, may be responsible for the endocrine disruption observed in these animals as well ***[EHP 117:797–802; Jobling et al.]***.

To help elucidate the complex relationships and interactions among the various types of endocrine disruptors (estrogenic, anti-estrogenic, androgenic, and antiandrogenic), the authors created statistical models based on 1) the chemicals’ known hormonal activities in recombinant yeast screen assays and 2) concentrations measured during an earlier national survey in effluent from U.K. wastewater treatment plants. The models also included hydrologic data to enable estimation of river-water chemical concentrations at specific sites and national survey data on the location and prevalence of feminized male fish. The statistical models first accounted for estrogenic effects observed in fish, then included effects associated with antiandrogens and other compounds.

The authors previously found a very strong correlation between the predicted steroid estrogen content of U.K. rivers and feminization in male wild fish. In the current study they focused on four specific traits of feminization: elevated plasma levels of vitellogenin (an egg yolk precursor protein normally produced only in females), feminized reproductive ducts, oocyte (egg cell) development in the testes, and the number of oocytes found in the testes. Once the main factors accounting for variation were identified, the researchers were able to distinguish the respective contributions of estrogens and antiandrogens to biologic responses and explore potential interactions.

Model estimates suggested that male fish exposed to the highest concentrations of estrogens or antiandrogens were the most likely to be feminized. However, chemical combinations were also important. Estrogens and antiandrogens acted additively with regard to oocytes in the testes, but estrogens appeared to antagonize effects of antiandrogens on feminization of the reproductive duct. The authors note that combined effects were not necessarily due to simultaneous exposure; for example, one compound could serve as an initiator early in life while another could act as a promoter later in life. On the basis of these analyses, the authors suggest that sexual disruption in male wild fish populations may be related to exposure to a combination of both estrogenic and antiandrogenic compounds, a relationship that may also hold true for humans.

## Figures and Tables

**Figure:**
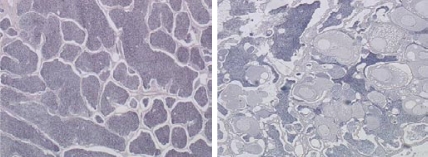
The micrograph on the left shows normal testis tissue from an adult male roach (*Rutilis rutilis*). On the right is the testis of a severely feminized male roach; the large circular inclusions are oocytes.

